# Effect of the COVID-19 Pandemic in the Prehospital Management of Patients with Suspected Acute Stroke: A Retrospective Cohort Study

**DOI:** 10.3390/ijerph19084769

**Published:** 2022-04-14

**Authors:** Natasza Blek, Lukasz Szarpak, Jerzy Robert Ladny

**Affiliations:** 1 Institute of Outcomes Research, Maria Sklodowska-Curie Medical Academy in Warsaw, 03-411 Warsaw, Poland; lukasz.szarpak@gmail.com; 2 Department of Neurology, Wolski Hospital, 01-211 Warsaw, Poland; 3 Research Unit, Polish Society of Disaster Medicine, 05-806 Warsaw, Poland; jerzy.r.ladny@gmail.com; 4 Research Unit, Maria Sklodowska-Curie Bialystok Oncology Center, 15-027 Bialystok, Poland; 5 Department of Emergency Medicine, Bialystok Medical University, 15-026 Bialystok, Poland

**Keywords:** stroke, prehospital, COVID-19, emergency

## Abstract

Acute Ischemic Stroke (AIS) can be successfully handled if it is noticed early in the prehospital setting and immediately diagnosed in the emergency department (ED). The coronavirus pandemic has altered the way health care is delivered and has had a profound impact on healthcare delivery. The effects could include prioritizing the prevention of COVID-19 spread, which could result in the discontinuation or deferral of non-COVID-19 care. We used the National Emergency Medical Service Command Support System, a register of medical interventions performed by emergency medical services (EMS) in Poland, to assess the impact of the COVID-19 epidemic across the Masovian Voivodeship on suspected stroke patients’ baseline characteristics, prehospital vital parameters, clinical and neurological status, emergency procedures performed on the prehospital phase and EMS processing times. Between 1 April 2019 and 30 April 2021, the study population included 18,922 adult suspected stroke patients who were treated by EMS teams, with 18,641 admitted to the emergency departments. The overall number of suspected stroke patients treated by EMS remained unchanged during COVID-19 compared to the pre-COVID-19 period; however, the average time from call to hospital admission increased by 15 min.

## 1. Introduction

Stroke is the second leading cause of death and the most common cause of long-term disability worldwide [1]. Additionally, Poland’s number of stroke patients is estimated to be between 60,000 and 90,000 per year [2]. However, between 2013 and 2018, figures from the National Health Fund show a 7.6 percent decline in the number of patients hospitalized for ischemic stroke (from 75,700 to 70,700) [3].

Acute Ischemic Stroke (AIS) is successfully managed when it is recognized early in the prehospital setting and diagnosed promptly in the Emergency Department (ED). Intravenous thrombolysis (IVT) has been recognized as an essential causal treatment for AIS to reopen a stenosed cerebral vessel and for which clinical benefit has been demonstrated in a time window of 4.5–9.0 h in numerous randomized controlled trials [4]. In addition, interventional catheter thrombectomy as an adjunct to IVT, used in cases of large vessel occlusion (LVO) in specialized neurovascular centers, is becoming increasingly important [5]. For functional outcomes after AIS, the time to recanalization is significant, as further brain tissue death threatens with each passing minute. For this reason, time management is of paramount importance in stroke care (“time is brain”) [6]. Given the proven high efficacy, however, still, too few patients have access to these forms of therapy; especially in rural regions, there is an underutilization of this patient group [7]. This is mainly due to delays in the prehospital phase, resulting in patients not reaching the clinic in time for causal treatment [8,9,10,11,12]. Therefore, the direction of treatment will depend on the correct prehospital diagnosis of the cause of the stroke. Thanks to prompt diagnosis, the implementation of appropriate procedures is crucial for a patient with a stroke, and time plays the most vital role here.

The pandemic of coronavirus disease has changed how health care is provided and has a significant impact on healthcare delivery. Effects may include prioritizing the prevention of the spread of COVID-19, which could lead to closing off or delaying non-COVID-19 care.

On 15 March 2020, the Polish Government declared the first limitations. Further measures were introduced on March 24, including the prohibition of non-essential travel, except work or home, SARS-CoV-2 monitoring operations, or “required day-to-day activities”.

On 20 March 2020, the Prime Minister announced an official outbreak in Poland. As a result, the Ministry of Health decided to transform 19 medical facilities into infectious hospitals—multi-specialized centers to provide patients with SARS-CoV-2 infections and significant comorbidities (e.g., COVID-19 patients with Acute Ischemic Stroke) [13].

Despite the hospital network organization, the pandemic has strongly affected the healthcare system’s balance and other facilities.

Guidelines on “Good practice in treating patients with suspected brain stroke for medical dispatchers and emergency medical services teams” were issued on 24 January 2019, by the Ministry of Health, in collaboration with State consultants in neurology and emergency medicine, to ensure proper prehospital treatment of suspected brain stroke and transportation to a specialist stroke unit [14].

Emergency medical services (EMS) are the initial point of contact for most stroke patients and are critical in the early detection of acute stroke. Immediate intervention of the rescue team should include examination of essential vital functions (pulse, respiration, blood pressure), measurement of glucose levels in capillary blood, and, if the patient’s state warrants it, administering basic medical life support to the patient (if applicable) [15].

The study’s initial objective was to analyze the diagnostic and therapeutic standards used in patients with cerebral stroke at the prehospital stage and the characteristics of stroke patients in general. However, because the COVID-19 pandemic occurred during the study, the objective was adjusted to include a comparison of pre- and post-pandemic cohorts. As a result, two time periods were investigated. We conducted this study to assess the impact of the COVID-19 epidemic across the Masovian Voivodeship on suspected stroke patients’ baseline characteristics, prehospital vital parameters, clinical and neurological status, emergency procedures performed in the prehospital phase, and EMS processing times.

## 2. Materials and Methods

### 2.1. Study Design

We conducted a retrospective cohort analysis of adult suspected stroke patients. Between 1 April 2019, and 30 April 2021, the study population included 18,922 adult suspected stroke patients who received prehospital care from EMS, of which 18,641 were admitted to the ED. Two time periods were investigated: 1 April 2019, to 31 March 2020 (pre-COVID-19) and 1 April 2020, to 31 March 2021 (COVID-19).

The statistics came from Poland’s largest voivodeship, the Masovian Voivodeship. The Masovian Voivodeship is Poland’s largest in terms of both territory and population. Additionally, it encompasses Poland’s capital, Warsaw. The region encompasses 35,579 km^2^. As of 31 December 2019, the district had a population of approximately 5.4 million. The voivodeship has 200 ground-based medical rescue teams stationed in 128 locations. Subjects were excluded if the information on age and basic vital parameters were missing.

Our work followed the STROBE (strengthening the reporting of observational studies in epidemiology) guidelines [16] and adhered to the Helsinki Declaration. The study was authorized by the Polish Society of Disaster Medicine’s Institutional Review Board (approval No. 10.03.2021.IRB).

### 2.2. Data Collection

Data were collected using the National Emergency Medical Service Command Support System, a register of ambulance dispatches and medical interventions done by emergency medical services (EMS) in Poland. Patients included in the study required an EMS dispatch due to neurological symptoms of cerebrovascular disease and had one of the following initial diagnoses—I60 (subarachnoid hemorrhage), I61 (intracerebral hemorrhage), I62 (other nontraumatic intracranial hemorrhage), I63 (cerebral infarction), and I64 (stroke not classified as hemorrhagic or infarct) according to the International Statistical Classification of Diseases and Related Health Problems revision 10 [17]. Following the collection of data from digital records, patients were de-identified. Gender, age, vital parameters, medical diagnosis, set of medical procedures performed, and medical treatment were all acquired from computerized medical records. The Polish healthcare system lacks a centralized, national stroke database. As a result, detailed information on the patients’ follow-ups was impossible to get. The analysis was limited to the prehospital management performed by the EMS crew and spans the time period from ambulance activation to patient transfer to the hospital’s emergency department.

### 2.3. Statistical Analysis

Statistical analysis was performed with the Statistical Package for Social Science (SPSS) version 27.0 software (SPSS, Inc., Chicago, IL, USA). Arithmetic means, medians, standard deviations, and range of variation (extreme values) were calculated for measurable variables. For qualitative variables, frequencies of occurrence (percentages) were calculated. All quantitative type variables studied were tested with the Shapiro–Wilk test to determine the type of distribution. Qualitative variables were compared between groups using the chi-square (χ^2^) test or Fisher exact method for small-sized samples. For the comparison of means, Welch’s *t*-test was used. An α = 0.05 level was used for all comparisons, and the resulting “*p*” values were rounded to 2 decimal places for statistically insignificant results and three decimal places for statistically significant results.

## 3. Results

From April 2020 to March 2021, medical emergency teams from the Mazovian region performed 386,764 patient encounters, of which 9544 were carried out on patients with suspected stroke, which constituted 2.47% of all EMS interventions in the pandemic period.

From April 2019 to March 2020, medical emergency teams from the Mazovian region performed 435,562 patient encounters, of which 9378 interventions were carried out on patients with suspected stroke—2.15% of all EMS interventions.

### 3.1. Baseline Characteristics

The overall baseline characteristics of our cohorts are presented in Table 1. Significant differences were found in sex, medical rescue team type, and scene. However, both groups were similar in terms of age, day, and day time of the intervention.

### 3.2. Vital Parameters and Clinical Status

There was no difference in preadmission vital parameters such as systolic and diastolic blood pressure, heart rate, respiratory rate, blood oxygen saturation, and clinical scores of GCS and RTS between the pandemic and pre-pandemic periods. The only difference was observed in the blood glucose level, which was increased during the pandemic period as presented in Table 2.

### 3.3. Emergency Procedures

Interestingly, statistically significant differences were observed regarding emergency procedures performed as summarized in Table 3.

Rates of peripheral vein cannulation dropped slightly during the pandemic period. On the contrary, rates of ECG, vital parameters, continuous monitoring, and oxygen therapy were increased.

In patients whose low blood pressure prompted the EMS head to initiate fluid therapy, 0.9% sodium chloride or polyelectrolyte fluid was administered. These activities were implemented in 8.5% of the patients in the pandemic period and 7.7% in the pre-pandemic period. In patients provided with qualified first aid by EMS members, drugs and other pharmaceuticals were administered orally/sublingually, intravenously, or inhaled. As far as the supply of agents influencing blood pressure is concerned, anti-hypertensive treatment was implemented in 8% of the patients in the pandemic period and 8.4% in the pre-pandemic period. Types of pharmaceuticals used are presented in Table 4.

The types of pharmaceuticals used to lower blood pressure are shown in Table 5 below.

### 3.4. Neurological Evaluation

This part of the analysis, shown in Table 6, covers the neurological symptoms faced by emergency medical teams during the examination and initial diagnosis. During the pandemic period, more patients presented bilateral miosis and poorer verbal response in GCS scores. Syncope on onset was less frequently witnessed.

### 3.5. Prehospital Time Intervals

As seen in Table 7, all prehospital time intervals were considerably delayed during the pandemic period. For example, the median time between call and contact with the patient was 3 min longer, whereas the period between contact and hospital admission was around 7 min longer.

## 4. Discussion

This study examines changes in the prehospital management of adult patients with acute stroke symptoms who utilized EMS in the Masovian Voivodeship prior to and during the COVID-19 pandemic.

In the first article published on neurological complications in COVID-19, Liu et al. reported six cases of acute cerebrovascular disease in their cohort of 214 patients in a retrospective case series study from Wuhan, China [18]. There is evidence that SARS-CoV-2 induces substantial systemic inflammation and increases thrombotic risk. Given the overwhelming data linking COVID-19 with thromboembolic events, it is reasonable to predict that stroke incidence would increase on the eve of a pandemic [19,20,21,22]. Oppositely, there could be associated factors that potentially reduce stroke incidence. Research published in The Lancet Neurology indicates that polluted air is one of the significant risk factors for stroke; therefore, a reduction could be potentially protective against stroke [23]. A striking reduction in pollution has been reported in multiple countries during the pandemic secondary to lockdown [24,25].

In our study, the overall number of suspected stroke patients treated with EMS during COVID-19 remained stable compared to the pre-COVID-19 period, while the average time from call to hospital admission increased by 15 min.

The COVID-19 epidemic has several consequences for stroke care, including a worldwide drop in stroke and cerebrovascular admissions [26]. However, the nation’s estimated number of acute stroke admissions has varied during the pandemic. In some countries, the estimated number of acute stroke admissions dropped by 50% and even 80% [27]. Numerous investigations have also established that individuals presenting with minor strokes and TIAs have experienced a drop during the epidemic [28,29]. In a smaller number of studies, the number of EMS referrals of stroke codes remained consistent throughout the COVID-19 pandemic lockdown [30,31].

Previous studies have presented possible explanations for the decline in acute stroke presentations. Most acute stroke patients with subtle symptoms stated that their delay in seeking medical attention was due to the fear of viral infection and transmission, given that SARS-CoV-2 is exceptionally infectious [32,33]. Fewer mild stroke or TIA admissions may be a social consequence of several reasons, including increased social isolation resulting in the absence of onset witnesses in cases of patients with resolving symptoms [34] as stroke symptoms are typically noted by another family member, friend, or community member prior to the patient recognizing them. This could also explain that syncope on onset was less frequently witnessed, and relatively more patients presented bilateral miosis and poorer verbal response in GCS scores, which could be the result of onset to contact delays and more severe clinical conditions of patients.

We observed a significant increase in blood glucose levels regarding patients’ vital parameters. This could be ascribed to weight increase, decreased physical activity, stress, and, in both types of diabetes, delayed diagnosis during lockdown and pandemic settings [35,36,37].

Interestingly, statistically significant differences were observed regarding emergency procedures performed. For example, rates of peripheral vein cannulation dropped slightly during the Pandemic period. On the contrary, rates of ECG, vital parameters continuous monitoring, and oxygen therapy were increased. This could indicate that crews were less likely to execute some standard procedures due to infectious concerns, but the patients were at a greater degree of acuity during the transfer. Unfortunately, the supply of anti-hypertensive drugs was unjustified in many cases as guidelines for prehospital management of stroke from the American Heart Association/American Stroke Association and the European Stroke Organization (ESO) state that even when systolic blood pressure is near 185 mmHg, which can increase door to needle time, paramedics’ immediate prehospital anti-hypertensive care poses a risk of unexpected decreases in blood pressure; as a result, elevated blood pressure care during the prehospital period should be avoided [15,38].

The causes for the increased prehospital delays remain unknown. Interestingly, a significant decrease in the number of EMS missions following the start of the COVID-19 outbreak was observed. Despite this, the EMS workload has increased—this could be due to increased hospital handover time, taking into account ED procedures for patients with a suspected infection, or as a result of higher ambulance decontamination demands [39]. Additionally, the requirement for thorough respiratory examination, COVID-19 screening, and the use of personal protective equipment may lengthen prehospital care duration [40].

### Limitations

Numerous limitations apply to this investigation. To begin, our study population included patients with the prehospital diagnosis of acute stroke; we did not confirm or follow whether they were ultimately diagnosed with stroke or a stroke mimic. Whether the diagnosis was verified, the same criteria should have been applied to patients experiencing acute stroke symptoms at the prehospital stage; consequently, this will not be a significant constraint in interpreting the results. Additionally, some patients may be missing, as we studied just the data provided by the EMS providers. Moreover, the time of symptom onset is critical in patients with acute stroke symptoms, but we did not include it since there were numerous missing data points on symptom onset in the records of EMS providers, making analysis impossible.

## 5. Conclusions

We conducted a retrospective cohort analysis using the National Emergency Medical Service Command Support System, a prospective register of medical interventions carried out by emergency medical services (EMS) in Poland to compare prehospital care delivered to suspected stroke patients during COVID-19 compared to the pre-COVID-19 period. When compared to the pre-COVID-19 period, the overall number of suspected stroke patients treated by EMS remained similar during COVID-19, but the average time from call to hospital admission increased by 15 min. Our findings lay the theoretical groundwork for the development of further guidelines to ensure adequate EMS for acute stroke, even in the event of an infectious disease outbreak.

## Figures and Tables

**Table 1:** Baseline characteristics.

		Pandemic (*n* = 9544)	Pre-Pandemic (*n* = 9378)	*n*	*p*	Test
Age—mean		72.8 (±15.7)	73.1 (±13.3)	17,818	0.21	Welch
Sex	Male	4043 (50%)	3771 (48%)	7814	**0.023**	Chi^2^
	Female	4102 (50%)	4111 (52%)	8213		
MRT type	B	7012 (73%)	6514 (69%)	13,526	**<0.001**	Chi^2^
	S	2532 (27%)	2864 (31%)	5396		
Scene	home	8673 (91%)	8125 (87%)	16,798	**<0.001**	Chi^2^
	public place	650 (6.8%)	1015 (11%)	1665		
	in traffic	42 (0.44%)	51 (0.54%)	93		
	workplace	146 (1.5%)	153 (1.6%)	299		
	school	7 (0.073%)	11 (0.12%)	18		
	farming	25 (0.26%)	23 (0.25%)	48		
Day	Monday	1359 (14%)	1263 (13%)	2622	0.29	Chi^2^
	Tuesday	1516 (16%)	1505 (16%)	3021		
	Wednesday	1372 (14%)	1454 (16%)	2826		
	Thursday	1357 (14%)	1337 (14%)	2694		
	Friday	1365 (14%)	1319 (14%)	2684		
	Saturday	1309 (14%)	1305 (14%)	2614		
	Sunday	1264 (13%)	1195 (13%)	2459		
Daytime	morning	5070 (53%)	4955 (53%)	10,025	0.74	Chi^2^
	evening	3219 (34%)	3154 (34%)	6373		
	night	1255 (13%)	1269 (14%)	2524		

**Table 2:** Vital parameters and clinical status.

	Pandemic (*n* = 9544)	Pre-Pandemic (*n* = 9378)	*n*	*p*	Test
Diastolic blood pressure in mmHg, mean	85.1 (±16.9)	84.6 (±16.6)	18,748	**0.037**	Welch
Systolic blood pressure in mmHg, mean	152 (±32.5)	152 (±32.4)	18,786	0.21	Welch
Blood glucose in mg/dL, mean	150 (±57.3)	145 (±55.0)	17,051	**<0.001**	Welch
Heart rate (/min), mean	86.2 (±21.1)	85.9 (±22.8)	18,698	0.32	Welch
Respiratory rate, mean	15.8 (±4.86)	15.9 (±4.96)	18,172	0.16	Welch
Blood oxygen saturation, mean	95.3 (±5.34)	95.4 (±5.02)	18,534	0.1	Welch
RTS score, mean	11.6 (±0.806)	11.6 (±0.767)	17,831	0.082	Welch
GCS, mean	13.1 (±2.58)	13.2 (±2.54)	18,604	0.069	Welch

**Table 3:** Emergency procedures.

		Pandemic (*n* = 9544)	Pre-Pandemic (*n* = 9378)	*n*	*p*	Test
ECG, *n*	not performed	6674 (70%)	6726 (72%)	13,400	**<0.01**	Chi^2^
	performed	2870 (30%)	2652 (28%)	5522		
IV cannulation, *n*	not performed	1891 (20%)	1571 (17%)	3462	**<0.001**	Chi^2^
	performed	7653 (80%)	7807 (83%)	15,460		
Vital parameters continuous monitoring, *n*	not performed	4715 (49%)	4908 (52%)	9623	**<0.001**	Chi^2^
	performed	4829 (51%)	4470 (48%)	9299		
Oxygen therapy, *n*	not performed	8706 (91%)	8642 (92%)	17,348	**0.02**	Chi^2^
	performed	838 (8.8%)	736 (7.8%)	1574		

**Table 4:** Types of pharmaceuticals used.

		Pandemic (*n* = 9544)	Pre-Pandemic (*n* = 9378)	*n*	*p*	Test
Antihypertensive treatment	not initiated	8742 (91.6%)	8628 (92%)	18,922	0.31	Chi^2^
	initiated	802 (8.4%)	750 (8%)	1552		
Fluid therapy	not initiated	8732 (91.5%)	8628 (92.3%)	17,386	0.053	Chi^2^
	initiated	812 (8.5%)	724 (7.7%)	1536		

**Table 5:** Types of anti-hypertensive medication used.

	Pandemic (*n* = 802)	Pre-Pandemic (*n* = 750)	*n*	*p*	Test
Captopril	562 (70%)	513 (68%)	1075	0.47	Chi^2^
Furosemide	58 (7.2%)	47 (6.3%)	105	0.45	Chi^2^
Urapidil	182 (23%)	190 (25%)	372	0.22	Chi^2^

**Table 6:** Neurological evaluation.

	Pandemic (*n* = 9544)	Pre-Pandemic (*n* = 9378)	*n*	*p*	Test
Localized weakness	3589 (38%)	3564 (38%)	7153	0.57	Chi^2^
Left-sided weakness	1832 (19%)	1891 (20%)	3723	0.094	Chi^2^
Right-sided weakness	1732 (18%)	1753 (19%)	3485	0.33	Chi^2^
Quadriplegia	51 (0.53%)	51 (0.54%)	102	0.93	Chi^2^
Isolated left lower extremity weakness	102 (1%)	90 (1%)	192	0.45	Chi^2^
Isolated right lower extremity weakness	111 (1%)	98 (1%)	209	0.44	Chi^2^
Isolated left upper extremity weakness	354 (4%)	316 (3%)	670	0.2	Chi^2^
Isolated right upper extremity weakness	392 (4%)	433 (5%)	825	0.086	Chi^2^
Anisocoria	92 (0.96%)	109 (1.2%)	201	0.18	Chi^2^
Bilateral miosis	522 (5.5%)	431 (4.6%)	953	**<0.01**	Chi^2^
Pupillary light reflex absent	151 (1.7%)	127 (1.5%)	278	0.19	Chi^2^
Aphasia	3152 (33%)	3002 (32%)	6154	0.14	Chi^2^
Convulsions	296 (3.1%)	275 (2.9%)	571	0.5	Chi^2^
Meningeal signs	142 (1.6%)	140 (1.6%)	282	0.94	Chi^2^
Vomiting	596 (6.5%)	534 (6%)	1130	0.13	Chi^2^
Syncope	1482 (16%)	1602 (18%)	3084	**<0.01**	Chi^2^

**Table 7:** Prehospital time intervals.

	Pandemic (*n* = 9544)		Pre-Pandemic (*n* = 9378)		*n*	*p*
	Mean	Median [Q25–75]	Mean	Median [Q25–75]		
Time from call to contact	17.2 (±18.1)	14.0 [9.00; 20.0]	12.9 (±7.56)	11.0 [8.00; 16.0]	18,920	**<0.001**
Time from contact to hospital admission	52.2 (±30.8)	45.0 [33.0; 63.0]	41.4 (±18.6)	38.0 [28.0; 51.0]	18,641	**<0.001**
Time from call to hospital admission	69.9 (±38.4)	61.0 [46.0; 83.0]	54.7 (±21.7)	51.0 [39.0; 66.0]	18,641	**<0.001**

## Data Availability

The data used to support the findings of this study are available from the corresponding author upon request.
